# Complete pathological response of colorectal peritoneal metastases in Lynch syndrome after immunotherapy case report: is a paradigm shift in cytoreductive surgery needed?

**DOI:** 10.1186/s12876-021-02084-x

**Published:** 2022-01-10

**Authors:** Marco Tonello, Floriana Nappo, Loretta Vassallo, Rosa Di Gaetano, Carla Davoli, Elisa Pizzolato, Ottavia De Simoni, Cristina Tassinari, Antonio Scapinello, Pierluigi Pilati, Fotios Loupakis, Sara Lonardi, Antonio Sommariva

**Affiliations:** 1 Surgical Oncology of the Esophagus and Digestive Tract Unit, Surgical Oncology Department, Veneto Institute of Oncology IOV-IRCCS, Via Gattamelata 64, 35128 Padua, Italy; 2 Medical Oncology 1 Unit, Department of Oncology, Veneto Institute of Oncology IOV-IRCCS, Padua, Italy; 3 Department of Surgery, Oncology and Gastroenterology, University of Padua, Padua, Italy; 4 Pathology Unit, Veneto Institute of Oncology IOV-IRCCS, Padua, Italy; 5 Transfusion Medicine and Immunohematology Unit, Flow Cytometry Lab, Castelfranco Veneto Hospital, AULSS 2, Veneto, Italy

**Keywords:** Cytoreductive surgery, Immunotherapy, Peritoneal metastasis, Colorectal cancer, Case report

## Abstract

**Background:**

We report the first case of a patient affected by peritoneal metastases from colon cancer, arising in the context of Lynch syndrome with pathological complete response. The patient was treated with immunotherapy and cytoreductive surgery. This paper discusses the implications of these novel therapies for the management of PM.

**Case presentation:**

A 50-year-old man affected by Lynch syndrome was referred to our institution for metachronous peritoneal recurrence of ascending colon adenocarcinoma. As a second-line treatment, he received Nivolumab therapy with stable disease. Patient underwent cytoreductive surgery with residual disease and a pathological complete response. Flow cytometry described a particular immune sub-population response. There was no evidence of disease progression after nine months.

**Conclusion:**

This is the first report of a Lynch patient affected by peritoneal metastases of colorectal cancer, treated with cytoreductive surgery (CRS) and resulting in a pathological complete response after immune checkpoint inhibitors treatment (ICIs). This case report may suggest that patients with peculiar immunological features could benefit from a tailored approach, since “classical” CRS paradigms may not effectively predict the clinical outcome. Further large-scale studies are needed to determine the correct operative management of such patients (tailored or “standard” CRS), defining the correct surgical timing and eventual discontinuation of ICI therapy after surgery.

**Supplementary Information:**

The online version contains supplementary material available at 10.1186/s12876-021-02084-x.

## Background

Peritoneal metastases (PM) represent a common and unfavorable evolution of colorectal cancer, estimated to develop in up to 19% of patients after radical surgery. PM has been estimated to be the cause of death in more than half of CRC patients [1]. Systemic chemotherapy is considered the mainstay treatment of patients with PM [2], even though PM is associated with shorter median survival compared to other metastatic sites (16.3 months for isolated PM) [3]. In selected cases, cytoreductive surgery (CRS) alone or combined with Hyperthermic Intraperitoneal Chemotherapy (HIPEC) has shown to improve the oncological outcomes of patients with PM [4, 5]. Selecting patients to be treated with CRS is pivotal; the peritoneal cancer index (PCI) and completeness of cytoreduction (CC) represent the most valuable selection factors for surgery [6]. There is increasing evidence that molecular cancer profiling could play a major role in the near future [7]. RAS/RAF mutations stratify long-term outcomes after CRS/HIPEC, improving patient selection for surgery. However, little data are available on the micro-satellite status relationship with the prognosis in PM patients.

In recent years, the introduction of immunotherapy in CRC cancer has shown promising results, especially in patients with a defective mismatch repair system (dMMR), which is detected in 15% and 4% of early stage and metastatic CRC, respectively [8–10]. However, the actual success rate of immunotherapy is limited to only about 30% of dMMR patients, meaning that only around 5% of all patients with CRC can really benefit from this approach [11]. The loss of MMR gene functions is usually through acquired mutations and is rarely caused by germline mutation in the context of Lynch syndrome. This syndrome, also known as hereditary non-polyposis colorectal cancer (HNPCC), is one of the most common hereditary cancer syndromes (1/279 estimated prevalence), accounting for about 3% of colon cancers [12, 13].

We report the first case of a patient with Lynch syndrome and peritoneal metastases from CRC who showed a pathological complete response at surgery after immunotherapy. This paper discusses the implications of these novel therapies in the surgical management of patients with PM.

## Case presentation

A 50-year-old man was referred to our institution for peritoneal recurrence after right hemicolectomy for CRC was performed in March 2017. The pathological report was consistent with mucinous adenocarcinoma pT3N0 (in 40 examined nodes), M0, stage IIa, no residual disease (R0), with a moderately differentiated grade (G2). KRAS, NRAS and BRAF genes were wild-type and microsatellite instability (MSH2 gene frameshift mutation) was present. He had no significant past medical history. However, his family history reported two relatives (father and maternal aunt) whose cause of death at the age of 40–45 was colon cancer; his mother, one sister, one brother and one daughter were alive without medical problems. After genetic consultation, the patient was diagnosed with Lynch syndrome (the brother and sister were tested but resulted negative).

**Fig. 1:**
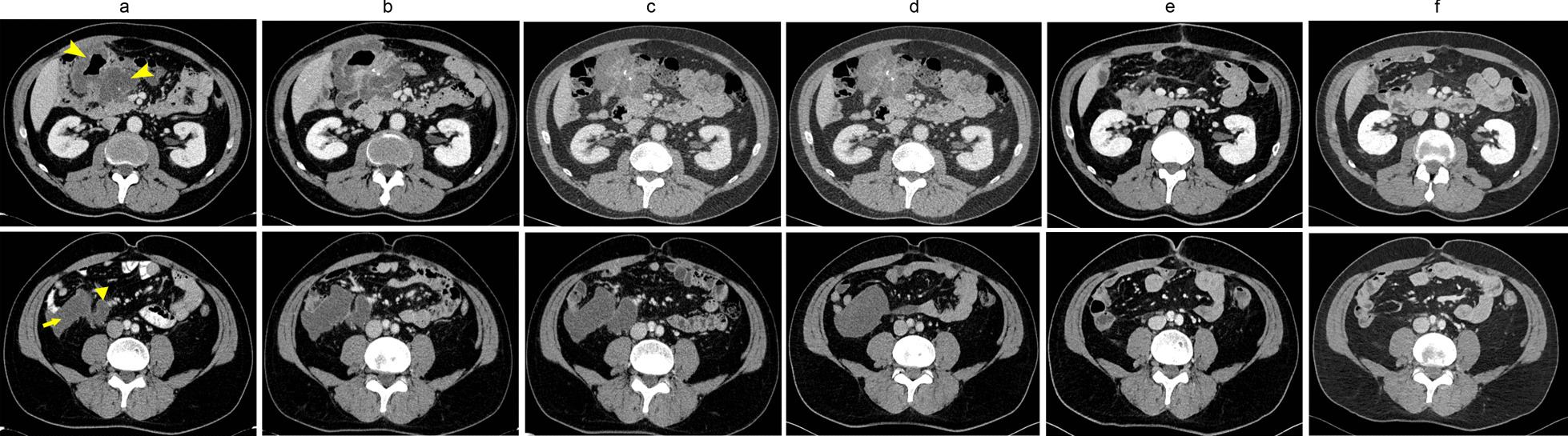
Title: Imaging (CT-scan) evolution of peritoneal metastases. Seriated CT-scan images: appearance of perianastomotic (top row) and right parieto-colic gutter region (lower row). **a** Recurrence diagnosis (May 2018), **b** after first-line (XELOX-Bevacizumab, July 2018), **c** after 2 months of Nivolumab (October 2018), **d** after 18 months of Nivolumab (February 2020), **e** three months after cytoreductive surgery (June 2020), **f** nine months after surgery (December 2020). Arrowheads: perianastomotic lesion, arrow: parieto-colic lesion, triangle: superior vessels nodal lesion

Fourteen months later (May 2018), a peritoneal and nodal recurrence (perianastomotic mass measuring 9 × 6 cm and right parietocolic gutter mass of 9 × 10 cm, superior mesenteric and portal vein nodes; Fig. 1a) was diagnosed, along with an increased CEA (9 ng/ml). The patient started systemic chemotherapy in June 2018 with a XELOX plus Bevacizumab regimen (Bevacizumab 7.5 mg/kg i.v. on day 1, Capecitabine orally twice daily 2,000 mg/m^2^/day on days 1–15 and Oxaliplatin 130 mg/m^2^ i.v. on day 1) at his referral hospital, suspended after 3 cycles due to gastrointestinal toxicities. The patient was referred to our center to evaluate second-line immunotherapy treatment. Given the current evidence and MSI status, we proposed off-label treatment with Nivolumab (240 mg every two weeks) in August 2018. At the first follow-up visit (December 2018), the CT-scan showed a RECIST 1.1 stable disease (10% increase in the diameter of both lesions) with normalization of CEA. At the following bi-monthly follow-up controls (physical examination, CT-scan and markers) the disease was stable (up to 15% reduction in both lesions at second control, minimal dimensional reduction in the followings follow-up controls; Fig. 1c–d). Immunotherapy was optimally tolerated and the patient reported an active life and returned to his full-time employment.

**Fig. 2:**
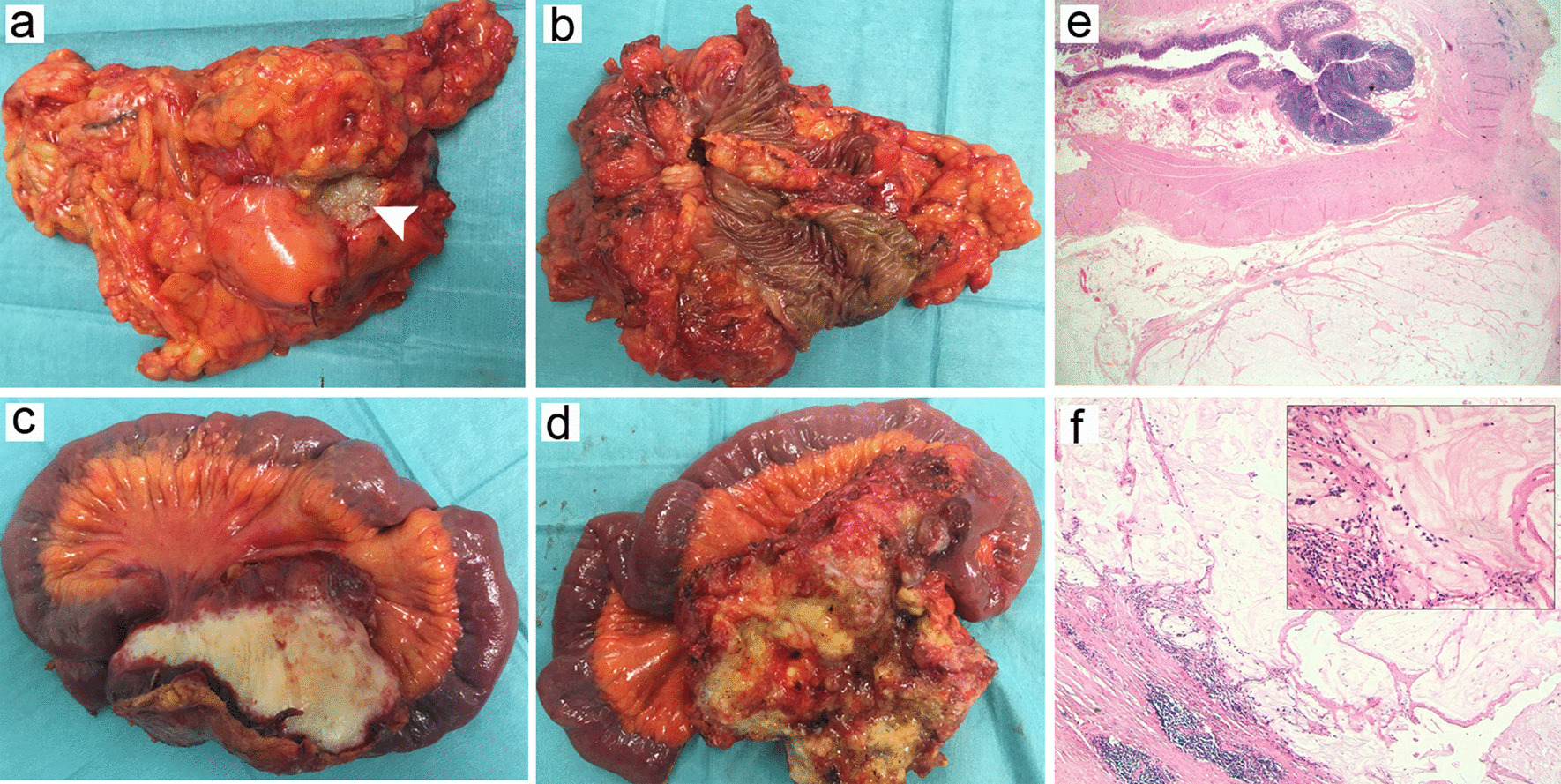
Macroscopical and pathological images of the resected specimen. **a** Perianastomotic recurrence (arrow-head: content of the mass through ex-vivo opening for pathological biopsy), **b** Intraluminal aspect of perianastomotic recurrence, **c** right parieto-colic mass with ileal resection, **d** content of the right parieto-colic mass, **e** Acellular mucin lakes in the muscularis propria and subserosal adipose tissue of the Ileocolic anastomosis (hematoxylin–eosin, ×20 magnification), **f** fibroinflammatory response associated with colloid material (hematoxylin–eosin, ×50 magnification) with detail of the lymphoplasmacytic infiltrate (hematoxylin–eosin, ×200 magnification)

Given the long disease-control interval, the case was discussed at a multidisciplinary meeting in February 2020 and the patient was scheduled for cytoreductive surgery. In March 2020, the patient underwent surgical exploration with resection of the previous ileocolic anastomosis (grossly involved by the tumoral mass) extending cephalad to 40 cm of the distal small bowel (the right parieto-colic mass was retracting the distal ileal mesentery and encapsulating the last branch of the superior mesenteric vein, SMV) (Fig. 2). After resection of the two main masses, we observed a residual yellowish firm mass of 0.5–1 cm in the upper site of the mesocolic excision confluent into the mesenteric vein root and a nodal enlargement beneath the head of the pancreas encircling the portal vein that would have required major resection (including pancreaticoduodenectomy and vascular reconstruction) to be safely removed. Several frozen section procedures of multiple biopsies on SMV nodes, perianastomotic and parieto-colic masses showed no evidence of tumor cells. Therefore, considering the high probability of a pathological complete response and the extension of the required resection, the surgical procedure ended with ileocolic anastomosis reconstruction and a possible macroscopic residual disease (completeness of cytoreduction grade: CC2).

The definitive pathological examination showed abundant mucinous deposits localised in all peritoneal biopsies and in the muscularis propria, subserosal adipose tissue and serosa of the intestinal wall, with the exception of mucosal and submucosal layers. Associated with mucin pools, there was significative fibroinflammatory response with a moderate amount of lymphoid aggregates, numerous plasma cells, histiocytes, foamy macrophages and multinucleated giant cells organised around the mucinous material. Newly formed blood vessels were also noted. No residual tumor cells were identified even after the almost complete sampling of surgical specimen (Fig. 2) (See Additional file 1: Additional Materials for pathology methods).

Given the distinctiveness of the case, we decided to perform flow-cytometry (FC) on a fresh sample of surgical specimen to describe the leukocyte population in the peritoneal metastasis. Leukocytes comprised 35% of nucleated cells and among these, 40% were lymphocytes. There was a high presence of B-cells (28%) among the lymphocytes, divided into naive B-cells (48%, CD19+/CD27−/SmIgD+ and activated plasma-cells (30%, CD38+). T-lymphocytes (60% of leukocytes) were present with a CD4/CD8 ratio of 1.8 with a moderate share (20% of the population) of activated cells (CD3+CD38+HLA/DR+. Due to the therapy received (Nivolumab), only 0.8% of lymphocytes resulted CD279 (PD-1 labelling marker) positive. On the other hand, T-lymphocytes showed a diffuse immune-modulating pattern. Actually, 45% of CD4 + T-lymphocytes expressed CD39 (NTPDase 1, a suppressive ectoenzyme) and CD152 (CTLA-4, another immune checkpoint). See Additional file 1: Additional Materials for FC data.

The postoperative course was uneventful. After multidisciplinary evaluation, we decided to suspend Nivolumab and the patient was scheduled for close follow-up. The patient was fully recovered one month after cytoreductive surgery, returning to his everyday activities, including full-time work. After nine months of close follow-up, repeated CT-scan (Fig. 1e, f) and blood analysis (including CEA) showed no disease recurrence.

## Discussion and conclusions

In recent years, the introduction of immunotherapy in CRC cancer has shown promising results and, in 2017, following reports of recent trials (KEYNOTE-164 and CHECKMATE-142) [14, 15] NCCN guidelines recommended pembrolizumab and nivolumab as second/third-line treatment in metastatic CRC patients. In most CRC patients with MSI, immunotherapies showed a good response rate and significant and durable disease control. However, not all patients selected for treatment respond and the pathways and mechanisms underlying the immune response are not fully elucidated. Alteration of MMR proteins, which are involved in the maintenance of genomic stability through the correction of DNA single nucleotide replication errors, are the basis of one of the most studied phenomena in immunological response [16]. Defective MMR may lead to microsatellite (short repeated DNA sequences) instability (MSI) and accumulation of errors, resulting in oncogenic transformation when errors occur in crucial coding regions [17]. Hypermutated and truncated proteins in MSI patients act as neoantigens to elicit an immune response in tumor-infiltrating lymphocytes (TILs) [18, 19]. Therefore, the rationale of immunotherapy is to promote the patient’s immune defense against tumor cells, mainly by avoiding activation-induced apoptosis in lymphocytes through the modulation of immune checkpoints (such as PD-1 and PD-L1 interaction) with drugs called immune checkpoint inhibitors (ICIs). [20–22]. Patients with metastatic CRC affected by Lynch syndrome are ideal candidates for immunotherapy, given that in 60–80% of cases, Lynch syndrome is caused by germline mutations in MLH1 and MSH2 or less frequently by MSH6, PMS2 and EPCAM mutations [18]. Pathological complete response has been observed in MSI or even Lynch syndrome patients treated with immunomodulating therapies, but at the present there is no evidence of complete response in stage IVc (PM) patients affected by PM. Experimental and clinical evidence showed that metastatic peritoneal nodules are relatively less responsive to standard systemic treatments. The reasons are not fully understood, but this phenomenon can probably be explained by relative hypovascularization and reduced drug diffusion in the peritoneal microenvironment.

Our report clearly illustrates that immunotherapy can be highly effective in CRC peritoneal metastases. Eventhough peritoneal recurrence has not be proven with biopsy before treatments begin, histological examination revealed the presence of well-known features of immune-mediated regression in other neoplasms, such as lymphoid aggregates, plasma cells and granuloma formation (activation), foamy macrophages (cell death), neovascularization and proliferative fibrosis (tissue repair) [23, 24]. These elements were associated with diffuse deposits of acellular colloid material in the tumor bed which is an already well-known CRC response to radiation and cytotoxic treatments, even in the absence of neoplastic cells. Mucin pool formation observed in our histological specimens (also called colloid response) has not been associated with ICI therapy. This could be a novel finding whose etiology is still not fully explained, even though neoplasm pathological features (mucinous adenocarcinoma) cannot be excluded as a causative factor [25, 26].

Important information was obtained from cytofluorimetric analysis. The analysis of immune infiltrating sub-populations showed a very high proportion of lymphocytes and plasma cells that are probably responsible for the pathological response. Very few regulatory T-cells were present among T-lymphocytes, whereas we observed an upregulation of CD39 (NTPDase 1, an ectoenzyme that degrades ATP to AMP with an anti-proliferative role) and CD152 (CTLA-4, an immune checkpoint-linked protein) positive T-lymphocytes; those features probably represent a counterbalance of the PD-1 pathway blockade with an immunosuppressive role [27–29], confirming the “effector” role of immunomodulation with Nivolumab in this case.

The complete pathological response observed after immunotherapy in a CRC patient with PM raises many questions that have to be considered in clinical management. Firstly, we do not know if CRS is really needed in this group of patients. Cytoreductive surgery has shown to give substantial clinical benefits in selected patients with PM from CRC, where limited disease and no residual disease can be obtained [5]. Although the additive role of HIPEC after CRS is still debated, several national guidelines recommend its use in experienced centers [30]. During the preoperative multidisciplinary discussion, we debated the association of HIPEC in the case of the complete surgical removal of the peritoneal metastases. Considering a possible detrimental effect of intraperitoneal cytotoxic agents in this particular patient [31, 32], we decided to administer HIPEC only in the case of peritoneal viable tumor cells (demonstrated with pathological intraoperative analysis). In the operating room, surgical procedures were tailored to this distinctive case, following a report of seriated pathological analysis conducted on different specimen and at different times of surgery. Our case suggests that a “paradigm shift” is needed when considering MSI patients with colorectal PM for surgery treated with immunotherapy. A complete pathological response confirmed by multiple intraoperative biopsies, should suggest a move from aggressive surgery (aimed at not obtaining macroscopic residual disease) to surgical exploration limited to performing biopsies and eventually, to palliate the symptoms when present. This approach requires a strong and updated multidisciplinary team, because a pathologist needs to know the patient’s history and novel treatment effects to provide the surgeon with quick and precise data, even with a limited-reliability frozen-section analysis. Unlike other metastatic sites, the confirmation of a pathological complete response in peritoneal nodules is clearly difficult to obtain without surgery. Laparoscopic exploration could be the best approach in the near future for cases with limited disease and a favorable environment (no peritoneal adhesion and easily accessible metastatic sites).

Considering the several open issues discussed in our case, it remains unclear if an incomplete CRS could represent a complementary means for eliminating potential immuno-resistant clones in the case of tumor heterogeneity in undetected microscopical residual neoplastic foci [33]. Moreover, indications to restart immunomodulating agents after a complete pathological response and incomplete surgery should be further addressed. Based on the evidence in other cancers on complete response after immunotherapy, we decided to stop treatment as it seems unlikely to obtain further therapeutic benefits and to avoid additional costs and the risk of side effects to the patient.

This is the first report of a Lynch patient affected by peritoneal metastases of colorectal cancer, treated with cytoreductive surgery and a pathological complete response achieved after checkpoint inhibitor treatment. Our case raises questions about new approaches that could be considered in MSI patients experiencing complete pathological response. Larger studies and more evidence are needed to draw any final conclusion on this topic. In case these initial findings would be confirmed in the next future, such patients may probably benefit more from a tailored surgical strategy (diagnostic/debulking surgery with respect to the “standard” cytoreductive surgery) and the discontinuation of ICI therapy after surgery. Considering this, further large-scale studies are needed to determine if ICIs in MSI patients with peritoneal metastases could change clinical approach.

## Supplementary Information

**Additional file 1:** Pathological and flow-cytometry analysis. Description of methods and results of pathological and flow-cytometry examinations, focusing on immunophenotype of CD4/CD8 T subsets.

## Data Availability

Relevant data (pathological and cytofluorimetric) are reported in the additional materials section. There are no datasets to be stored and made available since the study is a case report.

## Abbreviations

PM: Peritoneal metastases
CRS: Cytoreductive surgery
HIPEC: Hyperthermic intraperitoneal chemotherapy
RAS: Rat sarcoma gene
RAF: Rapidly accelerated fibrosarcoma gene
dMMR: Defective mismatch repair
MSI: Microsatellite instability
HNPCC: Hereditary nonpolyposis colorectal cancer
RECIST: Response evaluation criteria in solid tumors
FC: Flow cytometry
CTLA-4: Cytotoxic T-lymphocyte-associated antigen 4

## References

[1] Ferlay J, Colombet M, Soerjomataram I (2019). Estimating the global cancer incidence and mortality in 2018: GLOBOCAN sources and methods. Int J Cancer.

[2] Franko J, Shi Q, Goldman CD (2012). Treatment of colorectal peritoneal carcinomatosis with systemic chemotherapy: a pooled analysis of north central cancer treatment group phase III trials N9741 and N9841. J Clin Oncol.

[3] Franko J, Shi Q, Meyers JP (2016). Prognosis of patients with peritoneal metastatic colorectal cancer given systemic therapy: an analysis of individual patient data from prospective randomised trials from the Analysis and Research in Cancers of the Digestive System (ARCAD) database. Lancet Oncol.

[4] Van Cutsem E, Cervantes A, Adam R, Sobrero A, Van Krieken JH, Aderka D, Aranda Aguilar E, Bardelli A, Benson A, Bodoky G, Ciardiello F, Hoore A, Diaz-Rubio E, Douillard JY, Ducreux M, Falcone A, Grothey A, Gruenberger T, Haustermans K, Heinemann V, Hoff P, Köhne CH, Labianca R, Laurent-Puig P, Ma B, Maughan T, Muro K, Normanno N, Österlund P, Oyen WJ, Papamichael D, Pentheroudakis G, Pfeiffer P, Price TJ, Punt C, Ricke J, Roth A, Salazar R, Scheithauer W, Schmoll HJ, Tabernero J, Taïeb J, Tejpar S, Wasan H, Yoshino T, Zaanan A, Arnold D (2016). ESMO consensus guidelines for the management of patients with metastatic colorectal cancer. Ann Oncol.

[5] Quenet F (2018). A UNICANCER phase III trial of hyperthermic intra-peritoneal chemotherapy (HIPEC) for colorectal peritoneal carcinomatosis (PC): PRODIGE 7. J Clin Oncol.

[6] Hallam S, Tyler R, Price M, Beggs A, Youssef H (2019). Meta-analysis of prognostic factors for patients with colorectal peritoneal metastasis undergoing cytoreductive surgery and heated intraperitoneal chemotherapy. BJS Open.

[7] Schneider MA, Eden J, Pache B (2018). Mutations of RAS/RAF proto-oncogenes impair survival after cytoreductive surgery and HIPEC FOR peritoneal metastasis of colorectal origin. Ann Surg.

[8] Markowitz SD, Bertagnolli MM (2009). Molecular origins of cancer: Molecular basis of colorectal cancer. N Engl J Med.

[9] Issa JP (2004). CpG island methylator phenotype in cancer. Nat Rev Cancer.

[10] Koopman M, Kortman GA, Mekenkamp L (2009). Deficient mismatch repair system in patients with sporadic advanced colorectal cancer. Br J Cancer.

[11] Birendra KC, Hwang JJ, Farhangfar CJ, Jean Chai S (2017). Advances in immunotherapy in the treatment of colorectal cancer. AJHO.

[12] Yurgelun MB, Kulke MH, Fuchs CS, Allen BA, Uno H, Hornick JL (2017). Cancer susceptibility gene mutations in individuals with colorectal cancer. J Clin Oncol.

[13] Boland PM, Yurgelun MB, Boland CR (2018). Recent progress in Lynch syndrome and other familial colorectal cancer syndromes. CA Cancer J Clin.

[14] Le DT, Uram JN, Wang H (2015). PD-1 Blockade in tumors with mismatch-repair deficiency. N Engl J Med.

[15] Overman MJ, Lonardi S, Leone F, et al. Nivolumab in patients with DNA mismatch repair deficient/microsatellite instability high metastatic colorectal cancer: update from CheckMate 142. Gastroenterology Cancers Symposium 2017. [ASCO abstract 519]. J Clin Oncol. 2017;35

[16] Modrich P (2006). Mechanisms in eukaryotic mismatch repair. J Biol Chem.

[17] Duval A, Hamelin R (2002). Mutations at coding repeat sequences in mismatch repair deficient human cancers: toward a new concept of target genes for instability. Cancer Res.

[18] Brahmer JR, Tykodi SS, Chow LQ (2012). Safety and activity of anti–PD-L1 antibody in patients with advanced cancer. N Engl J Med.

[19] Schwitalle Y, Kloor M, Eiermann S (2008). Immune response against frameshift-induced neopeptides in HNPCC patients and healthy HNPCC mutation carriers. Gastroenterology.

[20] Gatalica Z, Snyder C, Maney T (2014). Programmed cell death 1 (PD-1) and its ligand (PD-L1) in common cancers and their correlation with molecular cancer type. Cancer Epidemiol Biomarkers Prev.

[21] Llosa NJ, Cruise M, Tam A (2015). The vigorous immune microenvironment of microsatellite instable colon cancer is balanced by multiple counter-inhibitory checkpoints. Cancer Discov.

[22] Xiao Y, Freeman GJ (2015). A New B7:CD28 Family Checkpoint Target for Cancer Immunotherapy: HHLA2. Clin Cancer Res.

[23] Cottrell TR, Thompson ED, Forde PM, Stein JE, Duffield AS, Anagnostou V (2018). Pathologic features of response to neoadjuvant anti-PD-1 in resected non-small cell lung carcinoma: a proposal for quantitative immune-related pathologic response criteria (irPRC). Ann Oncol: Off J Europ Soc Med Oncol/ESMO.

[24] G. Takayaki S et al. Successful treatment with nivolumab for lung cancer with low expression of PD‐L1 and prominent tumor‐infiltrating B cells and immunoglobulin. Thorac Cancer 2018 10.1111/1759-7714.12644 PMC5983224 29667757

[25] Nagtegaal ID, Glynne-Jones R (2020). How to measure tumour response in rectal cancer? An explanation of discrepancies and suggestions for improvement. Cancer Treat Rev.

[26] Dworak O, Keilholz L, Hoffmann A (1997). Pathological features of rectal cancer after preoperative radiochemotherapy. Int J Colorect Dis.

[27] Takenaka MC, Robson S, Quintana FJ (2016). Regulation of the T cell response by CD39. Trends Immunol.

[28] Li XY, Moesta AK, Xiao C (2019). Targeting CD39 in cancer reveals an extracellular ATP- and inflammasome-driven tumor immunity. Cancer Discov.

[29] Buchbinder EI, Desai A (2016). CTLA-4 and PD-1 pathways: similarities, differences, and implications of their inhibition. Am J Clin Oncol.

[30] Klaver CE, Groenen H, Morton DG (2017). Recommendations and consensus on the treatment of peritoneal metastases of colorectal origin: a systematic review of national and international guidelines. Colorectal Dis.

[31] Sargent DJ, Marsoni S, Monges G (2010). Defective mismatch repair as a predictive marker for lack of efficacy of fluorouracil-based adjuvant therapy in colon cancer. J Clin Oncol.

[32] Sinicrope FA (2010). DNA mismatch repair and adjuvant chemotherapy in sporadic colon cancer. Nat Rev Clin Oncol.

[33] Loupakis F, Maddalena G, Depetris I (2019). Treatment with checkpoint inhibitors in a metastatic colorectal cancer patient with molecular and immunohistochemical heterogeneity in MSI/dMMR status. J Immunother Cancer.

