# Supplementary material for: Complete pathological response of colorectal peritoneal metastases in Lynch syndrome after immunotherapy case report: is a paradigm shift in cytoreductive surgery needed?
Source: BMC Gastroenterol. 2022 Jan 10;22:17. doi: 10.1186/s12876-021-02084-x (PMC8751316; doi:10.1186/s12876-021-02084-x)

**Additional material**

Pathological analysis

Fresh surgical specimens of ileocolic anastomosis, terminal ileum resection, parieto-colic masses and multiple peritoneal nodules were submitted to the Pathology Department. A sample of a peritoneal lesion was placed in a saline solution for flow cytometry examination, while the remaining tissue was fixed in 10% buffered formalin and embedded in paraffin.

Flow-cytometry analysis

A sample of a peritoneal lesion was placed in a saline solution for flow cytometry examination. A single-cell suspension for flow cytometry (FC) analysis was prepared using GentleMACS Dissociator. The cell suspension was then resuspended and data acquisition was performed on a Navios Flow Cytometer. Sequential panels of monoclonal antibodies were used for the analysis of leukocytes and lymphocyte subpopulations.

Table 1. Immunophenotype of CD4 and CD8 T subsets

| % of Lymphocytes expressing flow-cytometric markers | CD4+ | CD8+ |
| --- | --- | --- |
| CD25 (IL-R2) | 9 | - |
| CD38 (cADPR) | 10 | 10 |
| CD25bright+, CD45RA-, CD127- (T reg) | 1 | - |
| CD152 (CTLA-4) | 45 | 45 |
| CD183+, CD196- (Th1) | 19 |  |
| CD183-, CD196+ (Th17) | 14 | 5.4 |
| CD183-, CD196- (Th2) | 19 | 10 |
| CD45RA-, CD197- (Tem) | 71 | 53 |
| CD45RA-, CD197+ (Tcm) | 1.5 | 1 |
| CD45RA-, CD197- (Tnaive) | 7.5 | 10 |
| CD45RA-, CD197- (Teff) | 20 | 36 |
| CD279 (PD-1) | 0.8 | 0.8 |
| HLA/DR | 9 | 10 |

Note: CD4+ lymphocytes are 54% and CD8+ 30% of all CD3+ cells. The CD4 and CD8 T subsets did not show the PD-1 antigen (CD279), but expressed the CTLA-4 (CD152). Mainly present were effector memory T (CD45RA-, CD197-) cells that migrate to tissues and display an immediate effector function. IL-2R: interleukin 2 receptor; cADPR: Cyclic ADP Ribose, Treg: T regulatory; CTLA-4: cytotoxic T-lymphocyte associated protein 4; Th: T helper lymphocytes; Tem: T effector memory, Tcm: T central memory; Teff: T effector; PD-1: programmed death - 1 protein.

**Figure 3** (additional materials)

Title: T and B lymphocytes flow-cytometry panel.

Note: nucleated cells (NC) were determined using the intercalating dyes DRAQ5. Leukocytes were identified and discriminated by CD45 antigen expression (Leukocyte Common Antigen). The lymphocytes gate was designed through lower Side Scatter values (SS, ordinate) and the higher intensity of the CD45 (CD45, abscissa). T and B lymphocyte populations were then defined and quantified by CD3 and CD19 antigen expression, respectively.


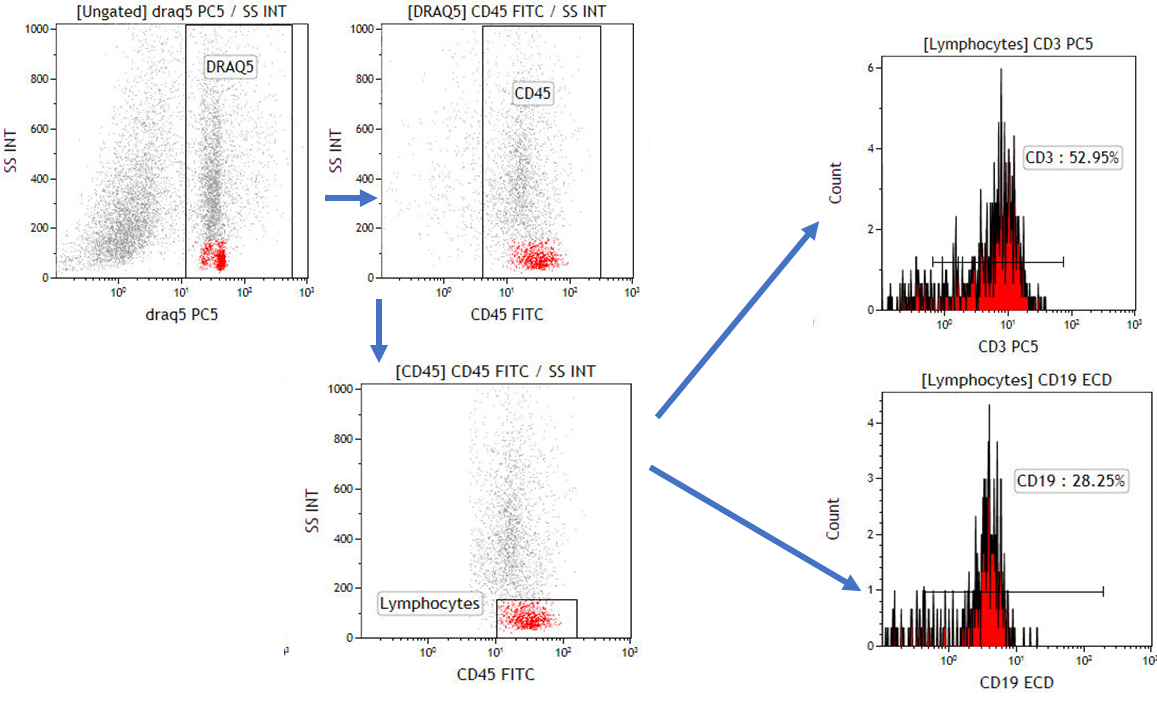

Supplement: Supplementary file 1 — Additional file 1: Pathological and flow-cytometry analysis. Description of methods and results of pathological and flow-cytometry examinations, focusing on immunophenotype of CD4/CD8 T subsets. [file 12876_2021_2084_MOESM1_ESM.docx]
